# Foot‐and‐mouth disease virus infection in the domestic dog (*Canis lupus familiaris*), Iran

**DOI:** 10.1186/s12917-021-02769-1

**Published:** 2021-02-01

**Authors:** Ryan A. Waters, Jemma Wadsworth, Valerie Mioulet, Andrew E. Shaw, Nick J. Knowles, Darab Abdollahi, Reza Hassanzadeh, Keith Sumption, Donald P. King

**Affiliations:** 1 The Pirbright Institute, Ash Road, GU24 0NF Woking, Surrey UK; 2 Iran Veterinary Organisation (IVO), Vali Asr Avenue, Tehran, Iran; 3 Food and Agriculture Organization for the United Nations (FAO), Rome, Italy

**Keywords:** Foot-and-Mouth Disease, Virus, FMD, FMDV, Heart, Cardiac, Dog, Myocardium, Iran

## Abstract

**Background:**

Foot-and-mouth disease (FMD) is a highly infectious viral disease, recognised to affect animals in the order Artiodactyla. The disease is rarely fatal in adult animals, however high mortality is associated with neonatal and juvenile infection.

**Case presentation:**

Five puppies died after being fed lamb carcases, the lambs having died during an outbreak of FMD in Iran. Following a post-mortem examination, cardiac tissue from one of the dead puppies was subjected to virus isolation, antigen ELISA, real-time RT-PCR, sequencing and confocal microscopy to assess the presence and characteristics of any FMD virus. The virological and microscopic examination of the cardiac tissue provided evidence of FMD virus replication in the canine heart.

**Conclusions:**

The data generated in this study demonstrate for the first time that FMD virus can internalise and replicate in dogs and may represent an epidemiologically significant event in FMD transmission, highlighting the dangers of feeding diseased animal carcases to other species. The reporting of this finding may also focus attention on similar disease presentations in dogs in FMD endemic countries allowing a better understanding of the prevalence of such events.

## Background

Foot-and-mouth disease (FMD) is an important transboundary animal disease, the aetiological agent of which is a picornavirus FMD virus (FMDV). The most commonly recognised signs of infection are vesicles on the feet and in the mouth, often associated with pyrexia. Acute myocarditis and death may also occur during infection of young susceptible animals. FMD directly impacts the livelihoods of farming communities in sub-Saharan Africa and large parts of Asia via reduced productivity and loss of draft-power, as well as imposing barriers on access to lucrative export markets for animals and animal products [1]. In FMD-free countries, outbreaks of the disease are feared as they can have an enormous impact on international trade. FMD is most frequently observed in domesticated livestock species within the order Artiodactyla (even toed ungulates) [2] such as cattle, sheep, goats, pigs and domestic water buffalo, and in wildlife including the African buffalo (*Syncerus caffer caffer*). Infection of non-artiodactyla species has been reported in natural and/or experimental settings such as in hedgehogs, rats, cats, kangaroos and dogs [3, 4], although evidence supporting natural infection in these species is often circumstantial. There are reports of FMD in dogs from the late 1800’s and early 1900’s when the disease was endemic in Europe [5, 6], however, no virological assessment was made of these cases. These observations, including blisters and cardiac abnormalities in animals which had contact with infected cattle, sheep and goats, prompted initial (unsuccessful) attempts by Loeffler, Frosch and Uhlenhuth in 1897 to demonstrate experimental FMD infection of dogs [6]. In contrast, three later studies conducted in Italy [7], Germany [6], and the UK [8] demonstrated experimental FMDV infection and disease via direct inoculation in dogs. A consistent feature of these reports was high mortality, with small vesicular lesions at the inoculation site being less consistent. In one of these studies, post mortem examination revealed lesions in cardiac tissue associated with necrosis and an inflammatory cell infiltrate [7]. Due to the rarity of these reports, FMD control programmes do not usually consider the potential role of natural FMDV infection in dogs. Indeed, dogs are only considered important in the context of spreading FMD as fomites, or by bringing material from infected farms to an uninfected farm by way of scavenging activity [9].

## Case presentation

**Fig. 1:**
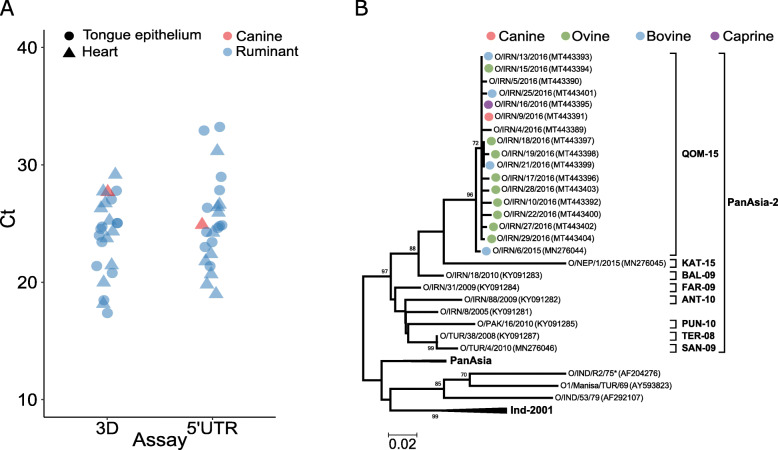
Genome analysis of foot-and-mouth disease virus (FMDV) detected in the canine heart. **a** Quantification of FMDV RNA genome using both 3D and 5′UTR real-time RT-PCR assays (quantity is proportional to 50 minus the Ct value on the y-axis) and comparison with epithelial and cardiac samples from suspected FMDV infected sheep and cattle submitted in the same batch of samples. The red triangle is the canine cardiac tissue. **b** Midpoint-rooted Maximum Likelihood phylogenetic tree employing the Hasegawa-Kishino-Yano nucleotide substitution model with a discrete Gamma distribution (5 categories [+ G, parameter = 0.3679)] (MEGA 7.0.26) based on the VP1-coding sequence obtained from samples submitted from outbreaks in Iran that occurred in 2016, including the dog cardiac sample (O/IRN/9/2016). Bootstrap values (1000 replicates) above 70 % are shown on the branches

In February-March 2016, cases of FMD due to serotype O were reported in Iranian provinces to the south and east of Tehran resulting in widespread disease in livestock, including reports of FMDV-associated mortality among young animals. In February, the Iran Veterinary Organisation (IVO) were informed that 2 puppies in Esfahan Province, and 3 in Kurdistan Province had become lethargic and weak 2 days after being fed carcases of lambs which themselves had died during an outbreak of FMD. During the following 1–2 days all 5 puppies died. Subsequent post mortem examination of these puppies by the IVO revealed lesions in heart tissue suggestive of FMD infection, and cardiac tissue from one case was collected and found to be positive for FMDV RNA via RT-PCR. This tissue was included in a batch of clinical samples collected from the region and submitted to the FAO World Reference Laboratory for Foot-and-Mouth Disease (WRLFMD), UK, for FMDV confirmation and characterisation. The canine origin of this sample was confirmed by using PCR amplification and sequencing of a fragment of the cytochrome c oxidase subunit 1 (COX1) gene [10] which demonstrated 99.69 % identity to GenBank sequence MN181403 (*Canis lupus familiaris* isolate CAN001). RNA was extracted from the submitted tissues, and FMDV RNA was detected by real-time RT-PCR targeting the 5′ untranslated and 3D polymerase regions [11]. The Ct values for IRN/9/2016 (3D, Ct 24.9; 5′ UTR Ct 27.7) were similar to those observed with the cattle and sheep tissue within the same batch of samples supporting the view that FMDV RNA detection in the canine cardiac tissue was not merely adventitious contaminating virus arising from sampling (Fig. 1a). The sample of canine cardiac tissue (designated IRN/9/2016) was first subjected to virus isolation on a susceptible monolayer of primary bovine thyroid cells (BTy) [12] and the resulting isolate tested positive for serotype O using a serotype-specific antigen ELISA.

Complete FMDV genomes (8189 nucleotides) obtained from both the canine heart sample and isolate IRN/9/2016 (GenBank accession numbers MT944980 and MT944981 respectively) were determined using next-generation sequencing [13]. Phylogenetic analyses of the 1D (VP1) encoding region of this sequence [14] confirmed that IRN/9/2016 was a serotype O virus characterised within the O/ME-SA/PanAsia-2^QOM − 15^ lineage closely related to FMDVs circulating concurrently in the region (Fig. 1b). Across the complete genome, comparison to the most closely related sequence, O/IRN/16/2016 (infected heart tissue from a goat and the subsequent isolate, GenBank accession numbers MT944982 and MT944983 respectively) highlighted six amino acid substitutions: three within the leader protein (A13T, I17F, S19T), one in 2C (T248I), one in 3A (V146A) and one in 3D^pol^ (E148G). The 3A region of the FMDV genome has previously been associated with host specificity, with deletions present in the 3A region of isolates of swine origin found to be attenuated in cattle. Interestingly, a reverse mutation in the same position (I248T) has previously been observed in an FMDV isolate experimentally adapted to the guinea pig which may indicate importance in this site for species specific tropism [15]. Moreover, the three close mutations in the leader protein may also be significant given the role of the leader protein can have in dampening the host innate and adaptive immune responses [16]. However, further work is required to determine the significance of the mutations observed here with regards to adaptation to replication in dogs.

**Fig. 2:**
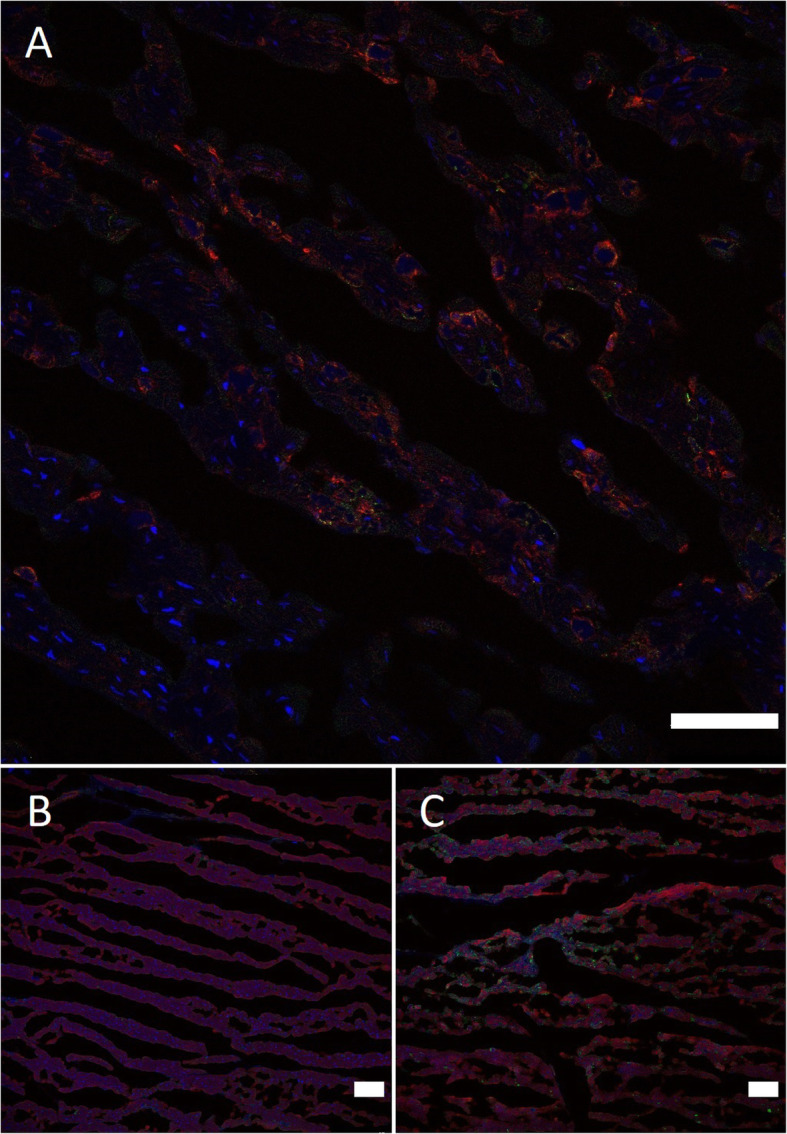
Confocal microscopic images demonstrating the presence of foot-and-mouth disease virus (FMDV) proteins in the canine cardiac tissue. **a** Detection of FMDV NSP (green), and FMDV capsid (red). Cell nuclei were stained with 4′,6-diamidino-2-phenylindole (DAPI, blue). **b** An area of canine cardiac tissue stained with phalloidin (red) demonstrating a normal staining pattern and no FMDV NSP detected. **c** An area of cardiac tissue stained with phalloidin demonstrating a disturbance in cardiac myocyte morphology with associated FMDV NSP detection in green. White scale bar = 50 µm

The presence of FMDV proteins and microscopic cardiac damage was imaged using confocal microscopic examination. The cardiac tissue, which had been stored in a 50:50 v/v PBS:glycerol solution at -20 °C, was placed in PBS at 4 °C overnight to remove the glycerol and the following day was frozen in a cryomould containing optimal cutting temperature (OCT) compound. Tissue sections were immunolabelled using a mouse IgG2a monoclonal antibody (mAb), 2C2, targeting the FMDV non-structural protein 3A and a mouse IgG1 mAb, IB11, previously described as targeting the FMDV capsid [17]. Primary immunolabelling was detected using fluorophore conjugated polyclonal secondary antibodies specific to the primary mAb isotype. Fluorophore conjugated phalloidin was used to highlight the structure of cardiac myocytes by binding to the intracellular F-actin. The slides were examined using an SP8 Leica confocal microscope. Microscopic examination detected both structural and non-structural FMDV proteins, providing evidence of viral replication in the canine cardiac tissue (Fig. 2a). Furthermore, phalloidin staining revealed that areas of FMDV protein labelling were associated with disruption of cardiac myocyte morphology (Fig. 2c), whereas areas of the heart tissue where the cardiac myocytes appeared normal had no areas of FMDV labelling (Fig. 2b).

## Discussion and conclusions

In summary, the data presented here support the conclusion that canine fatalities occurred as a result of FMDV infection in puppies which had fed upon infected carcasses in an outbreak of serotype O FMDV in Iran. This represents the first description of natural infection of dogs by FMDV. Specifically, evidence is provided to show that FMDV can infect and damage the cardiac myocytes and was the cause of death in at least one of these animals. The wider epidemiological importance of this finding is unknown. However, wider awareness of this case report should ensure that a provenance of FMDV infection is given greater consideration in scenarios entailing canine deaths associated with FMD outbreaks in endemic countries. Importantly there is a clear risk of feeding young dogs the carcases of animals which have been infected with FMDV, and therefore this practice should be avoided.

## Data Availability

The sequencing datasets generated and analysed during the current study are available in GenBank as per the accession numbers referenced in the text: MT944980 - https://www.ncbi.nlm.nih.gov/nuccore/MT944980 MT944981 - https://www.ncbi.nlm.nih.gov/nuccore/MT944981 MT944982 -https://www.ncbi.nlm.nih.gov/nuccore/MT944982 MT944983 - https://www.ncbi.nlm.nih.gov/nuccore/MT944983 The raw confocal images, and the real-time RT-PCR data are available from the corresponding author on reasonable request.
